# Senecavirus a can replicate in apical-out porcine intestinal organoids and induce stress granules and innate immune response

**DOI:** 10.1080/21505594.2025.2548623

**Published:** 2025-08-24

**Authors:** Ying Wang, Zhiying Wei, Jiaxuan Li, Xiaona Wang, Yanping Jiang, Wen Cui, Zhifu Shan, Lijie Tang

**Affiliations:** a College of Veterinary Medicine, Northeast Agricultural University, Harbin, China; b Northeast Science Observation Station for Animal Pathogen Biology, Ministry of Agriculture and Rural Affairs, Harbin, China

**Keywords:** SVA, intestinal organoids, replication, stress granules, 3D culture

## Abstract

Senecavirus A (SVA) causes clinical blistering and ulcerative lesions resembling foot-and-mouth disease (FMD), often with mixed infections that exacerbate the disease and impact pig industry development. SVA has been demonstrated to induce diarrhea, dehydration and mortality in piglets. However, the underlying mechanisms of SVA-related intestinal infections remain underexplored. In this study, a three-dimensional cultured apical-out porcine intestinal organoid model was constructed, comprising a variety of cell types, including intestinal stem cells, enterocytes, goblet cells, proliferative cells, Paneth cells and enteroendocrine cells. The model demonstrated SVA susceptibility in intestinal epithelial cells through cytopathic effects, viral detection, and replication. The results revealed an infection sequence from enterocytes to enteroendocrine cells, Paneth cells and intestinal stem cells and ultimately to proliferating cells, which identified enterocytes as the primary SVA targets. The presence of stress granules was observed at 4 hours post-infection (hpi), with a notable decline over time, reaching near-disappearance at 20 hpi. At this stage, an innate immune response was evident, with significant upregulation of the interferon IFN-α, the interferon-stimulated gene ISG-15, OAS1, OAS2, the signal transducer and activator of transcription 1 (STAT1), the mucosal immunity gene Muc2, and the cytokine IL-6, which appeared to limit further SVA infection. This study elucidates the infection pattern of SVA in intestinal epithelial cells and reveals the mechanism of interaction. It offers insights for controlling secondary infections.

## Introduction

Senecavirus A (SVA) is an emerging picornavirus that cause blistering and ulcerative diseases in swine. Its clinical symptoms resemble those of foot-and-mouth disease and swine vesicular disease. SVA can affect pigs of all ages [1], causing symptoms including anorexia, lameness, lethargy, and fever, which may result in death in severe cases. The SVA was discovered accidentally in 2002 in the United States during adenovirus research [2]. The cases have been reported across several countries, including Australia [3] and Brazil [4], resulting in severe economic losses. Since the SVA strain was first isolated in China in 2016, its prevalence has increased, spreading across regions and posing a serious threat to the pig industry [5]. Therefore, a thorough investigation into the biological characteristics and infection mechanisms of SVA is essential to effectively control its spread.

SVA is the sole representative of the genus Senecavirus within the picornavirus family. Its genome is a single-stranded, positive-sense RNA, approximately 7.4 kb long. This genome encodes a single polyprotein, which is cleaved to produce the structural proteins VP1, VP2, VP3, and VP4, along with the non-structural proteins 2A, 2B, 2C, 3A, 3B, 3C, and 3D, among others. The viral particles of SVA are small, with diameters ranging from 27 to 30 nm and a molecular weight around 30 kDa [6]. The viral capsid exhibits a classical icosahedral symmetry and lacks a capsid envelope. Recently identified SVA strains can cause significant blistering and ulcerative lesions on the snouts and hoof crowns of swine. Clinical symptoms in pigs under one week of age include weakness, hypersalivation, skin congestion, diarrhea and sudden death. The mortality rate of piglets within the first 4 days after birth can be as high as 70% [1].

SVA exhibits a strong preference for epithelial tissues and can be detected in various organ systems. Significant pathological alterations are primarily observed in the spleen, lungs, lymph nodes, and small intestine [7]. Notably, the kidneys display signs of hemorrhage and pronounced hyperplasia of the uroepithelium in the renal pelvis, ureters, and bladder, with localized lymphocytic and mononuclear cell infiltration. Pulmonary symptoms include edema and congestion, along with lymphadenopathy. Mesenteric edema, atrophy, fusion, and even necrosis of the small intestinal villi have also been observed [8]. The apical intestinal epithelial cells of piglets with clinical signs of diarrhea show necrosis and vacuolization [1]. The shedding of SVA particles have been detected in fecal samples from a numerous symptomatic piglets, as well as fattening pigs with blistering lesions [9]. This has enabled the simultaneous detection of common porcine enteroviruses, including circoviruses [10]. These findings suggest that SVA may often co-infects with other porcine viruses, indicating that the intestine is a critical site for SVA infection. Therefore, it is essential to establish appropriate in vitro models of the intestine to study SVA infection mechanisms.

Currently, there is a paucity of in vitro studies focusing on SVA infection in the porcine intestinal tract. Most existing research relies on porcine testis (ST), porcine kidney (IBRS-2 and PK-15), and baby hamster kidney (BHK-21) cell lines [11]. However, these models are insufficient for studying pathogen infections in intestinal epithelial cells. Consequently, this study aims to establish a more physiologically relevant in vitro infection model that more closely reflects the composition and functional state of intestinal cells in vivo, thereby advancing the understanding of SVA infection mechanisms in intestine. The emergence of porcine intestinal organoid technology provides a promising approach to address these challenges and offers a unique platform for analyzing host-pathogen microbial interactions in vitro environment.

Cultured organoids rely on extracellular matrix hydrogel (Matrigel) to form specialized three-dimensional (3D) structures, that mimic natural tissue microenvironment. However, this can create pseudo-cavities and basal topologies, hindering within the organoids, thus preventing direct access to the apical side of the intestinal epithelium [12]. To address this, researchers have developed various derivative models for studying intestinal pathogens and to facilitate viral infections. Some studies have preserved the original 3D structure of intestinal organoids and facilitated infection by directly blowing the intestinal organoids up and down with a pipette tip, followed by mechanical segmentation and incubation of the resulting fragments with viruses [13,14]. This method causes mechanical disruption of the intestinal organoids, leading to greater damage and loss of epithelial polarity. Alternatively, pathogenic microorganisms can be microinjected directly into the lumen of the intestinal organoids [15]. However, the technique is demanding and requires stability and extensive experience on the part of the technician [16]. Some studies have suggested that intestinal organoids can be modified into a 2D monolayer structure, allowing for the addition of viral particles to facilitate infection [17]. However, this method limits the ability to perform relevant infection studies on the outside of the substrate. Therefore, researchers have explored the establishment of a gas-liquid model of organoid monolayer cell culture using Transwell plug systems, which allow infection studies on the polar apical side, addressing the inaccessibility of the basolateral surface of organoids [18,19]. However, the 2D intestinal organoid model fails to replicate the 3D structure. This limitation results in the loss of critical cell-cell interactions, and deviates significantly from actual intestinal crypt structure and epithelial cell structure. Consequently, researchers have developed an apical-out intestinal organoid model. In 2019, Co et al. used a suspension culture system to reverse the polarity of human intestinal organoids, thereby establishing an innovative apical-out intestinal organoid model [20]. Li et al. applied this method to create an apical out model of porcine intestinal organoids and introduced the porcine transmissible gastroenteritis virus (TGEV) [21]. The results demonstrated that these apical-out porcine intestinal organoids are susceptible to viral infection and can mount an antiviral immune response. These porcine intestinal organoids serve as ideal models to study porcine enterovirus-host interactions

To further elucidate the mechanisms underlying host-pathogen interactions in this model, it is important to examine how host cells respond to SVA-induced cellular stress during infection. In the progress of viral infection, viral proteins or nucleic acids, which are recognized as foreign substances, have the potential to elicit stress responses in host cells. These responses may include autophagy, the formation of stress granules (SGs), apoptosis, and pyroptosis. SGs serve as storage sites for mRNA, aiding in regulation of its translation, localization, and degradation [22]. SGs respond to various environmental stresses and viral infections, representing a key pathway for host cells reactions to pathogens. The G3BP1 protein, a member of the RNA-binding protein family, is primarily located in the cytoplasm and exhibits a range of biological activities. It plays a crucial role in diverse cell signaling pathways and RNA metabolism, making it a vital component of SGs [23]. SVA viral infections induce cellular stress and modulate gene expression by influencing mRNA translation, localization, and degradation [24]. A significant correlation exists between SGs and SVA, particularly in SVA infection, replication is inextricably linked to SG dynamics [25].

Accordingly, the present study investigated the infection and proliferation characteristics of SVA using apical-out porcine intestinal organoids culture system, which elucidates the infection pattern of SVA in intestinal epithelial cells and the identification of the interaction mechanisms between low permissiveness cells and SVA. It offers insights for controlling secondary infections.

## Materials and methods

### Viruses and animals

The SVA LJ-2021 strain was isolated and preserved by our laboratory for subsequent analysis. The pigs were procured from a commercial pig farm in Harbin. All experimental procedures and animal care protocols were approved by and conducted in accordance with the guidelines set forth by the Laboratory Animal Ethics Committee of Northeast Agricultural University (protocol approval number **NEAUEC2022 03 15**) and complied with the Regulations for the Administration of Affairs Concerning Experimental Animals as mandated by the State Council of the People’s Republic of China.

### Porcine intestinal organoid culture: Isolation, passaging, freezing and thawing

The pigs were euthanized by intramuscular injection of Zoletil (BN 8N5TA) at a dose of 12 mg/kg and cervical dislocation was performed after the pig was sedated. The crypts of small intestine were isolated from the jejuna of six seven-day-old sows (Three technical repetitions). Briefly, a 5 ~ 10 cm segment of jejunum was collected and opened longitudinally, after which the intestinal sections were cut into 2 mm segments. The intestinal debris was washed with PBS a total of five times until the cleaning solution appeared clear. The intestinal debris was incubated in 10 mL of cell dissociation reagent (StemCell 07,174) for 20 minutes on a shaker at room temperature. Removal of supernatant after natural settling of the intestinal debris, pieces were added to 10 mL of pre-cooled PBS containing 0.1% BSA and the intestinal debris was gently agitated. The supernatant was then placed on a 70 µm filter after natural settling of the intestinal debris. Subsequently, the supernatant containing the crypts was centrifuged at 1200 rpm for a duration of 5 minutes. The 50 crypts were resuspended in 25 μL of Matrigel (Corning 356,234) and 25 μL of organoid growth medium (StemCell 06,010), and 50 μL of crypt suspension was carefully pipetted into a 48-well cell culture plate [17]. The 48-well cell culture plate was incubated at 37°C for 15 min and 200 μL of organoid growth medium was added to each well and incubated at 37°C, 5% CO2 for the duration of the experiment. The medium was replaced every 3 to 4 days.

In order to passage on porcine intestinal organoids, they were collected using pre-cooled PBS and subjected to centrifugation at 1500 rpm for a period of 5 minutes to remove majority of the Matrigel. The organoids were incubated with 0.25% EDTA-trypsin (Beyotime, C0201) at 37°C for 5 minutes. The digestion was terminated by the addition of DMEM/F12 (Cellmax, CGM105.05) containing 10% FBS (Animal Blood Ware, FBS0500S) to obtain a single cell suspension. The cells were subjected to centrifugation at 1500 rpm for 5 minutes. The cells were resuspended in Matrigel and organoid growth medium in a 48-well cell culture plate. The 48-well cell culture plate was incubated at 37°C for 15 min and 200 μL of organoid growth medium was added to each well and incubated at 37°C, 5% CO2 for the duration of the experiment. The medium was replaced every 3 to 4 days.

A cryopreservation solution consisting of 90% FBS and 10% DMSO was prepared and transferred into 2 ml cryovials, each containing 1 × 10^6^ to 5 × 10^6^ cells. The tubes were placed in a Programed Cryopreservation Container (Beyotime, FCFC012) at − 80°C for a minimum of one day. Subsequently, the frozen cryovials were transferred to a tank of liquid nitrogen for storage.

To thaw the organoids, the frozen organoids were rapidly heated in a water bath maintained at 37°C. The organoid mixture was washed with 10 mL of DMEM/F12 and cultured with Matrigel and organoid growth medium in a 48-well cell culture plate at 37°C with 5% CO2. The culture medium was replaced with fresh medium after 6 to 8 hours of resuscitation and subsequently every 3 days.

### Development of apical-out porcine intestinal organoids

Porcine basal-out intestinal organoids cultured in Matrigel for a period 3 to 5 days were subsequently collected using pre-cooled PBS. A solution of 5 mM cold EDTA (Beyotime, C0196) was added, and the mixture was placed on a rotating platform for 1 h at 4°C. The organoids were subjected to centrifugation at 1500 rpm for 5 min at a temperature of 4°C, following which they were washed twice with DMEM/F12. Y-27632 Mechanical support in the polarity reversal of intestinal organoids. The organoids were cultured for approximately three days in ultralow-attachment 24-well tissue culture plates in organoid growth medium containing 10 μM Y-27632 (Cell Signaling Technology 13,624) at 37°C, 5% CO2 in an incubator.

### Indirect immunofluorescent staining of organoids

The organoids were collected in a 1.5 mL tube and fixed with 4% paraformaldehyde for a period of 1 ~ 2 hours (up to 10 hours) at 4°C. Following the washing of the organoids pellet with PBS, it was permeabilized with 200 μL of 0.25% Triton X-100 (Sigma-Aldrich, 9002–93–1) for a period of 20 ~ 30 minutes at room temperature. The pellet was then washed three times with PBS and spined down at 1200 rpm for 5 min. To prevent nonspecific binding, the pellet was resuspended with 200 μL of 5% BSA for 1 h at room temperature.

The primary antibodies were diluted at a ratio of 1:50 with 5% BSA, including mouse anti-β-catenin monoclonal antibody (Abcam, Ab22656), rabbit anti-Lgr5 polyclonal antibody (Novus, NBP1–28904), rabbit anti-villin monoclonal antibody (Boster, PB9457), rabbit anti-Muc2 monoclonal antibody (Abcam, Ab272692), rabbit anti-Ki67 monoclonal antibody (Boster, PB9026), rabbit anti-Lyz polyclonal antibody (Servicebio, GB11345–100), rabbit anti-ChgA polyclonal antibody (Servicebio, GB111316–100), rabbit anti-ZO-1 polyclonal antibody (Invitrogen, WE323293), mouse anti-SVA N protein monoclonal antibody (prepared and preserved in our laboratory), and rabbit anti-G3BP1 polyclonal antibody (Proteintech 13,057–2-AP). These antibodies were incubated with the organoids at 4°C overnight. The pellet of organoids were washed three times with PBST.

The secondary antibodies were diluted with 5% BSA and included goat anti-rabbit IgG TRITC (Sigma-Aldrich, AP132) and goat anti-mouse IgG FITC (Sigma-Aldrich, AP124). The organoids were incubated with the afore mentioned antibodies for 1 h at room temperature in the dark. Following three washes of the pellet of organoids three times with PBST, it was resuspended in DAPI (Biotopped, Top0223) diluted at 1:1000 with PBS for 15 min at room temperature. The pellet of organoids were washed twice with PBS and then centrifuge at 1200 rpm for 5 min and transferred to the center of the slide, followed by the addition of Antifade Mounting Medium (Beyotime, P0126). Vacuum grease was filled into a 10 mL syringe and vacuum grease was applied to the corners of the slides as spacers. A coverslip were placed on top. The images were captured using a confocal microscope.

### Virus infection on apical-out porcine intestinal organoids

Apical-out porcine intestinal organoids were harvested from the culture suspension and inoculated with the SVA LJ-2021 strain (MOI = 6) for a period of 2 h at 37°C. Subsequently, the residual viral material was removed, and the organoids were washed three times with DMEM/F12. The organoids were transferred to the organoid growth medium for incubation and observation at 37°C and 5% CO2 in an incubator.

### RNA extraction and quantitative real-time PCR

The SVA-infected organoids were collected and subjected to total RNA extraction kit for cells (Fastagen 220,011). The total RNA was then employed in the preparation of cDNA with the Thermo Scientific RevertAid RT kit (Invitrogen, K1691). To assess the SVA virus copy number and the relative quantities of the genes, RT-qPCR was conducted using SYBR Green qPCR mix (Monad, MQ00801S) as references with GAPDH and Actin Beta (ACTB) as the internal control. The relative expression of each target gene in the cell was calculated according to Equation 2^−∆∆CT^ for differential level analysis. The laboratory has established a standard curve of SVA virus copy number, the formula is: Lg (virus copy number)/μL = −3.0216 (Ct value) +39.11452, the Ct value will be substituted into the formula to obtain the specific value of virus copy number. “Copies/μL” denotes the concentration of viral nucleic acid in the nucleic acid replicate solution of the corresponding sample following nucleic acid extraction and RT-qPCR. This measurement is utilized to quantify the viral load across different samples in a standardized manner. The primer sequences employed in this study are listed in Table 1.

**Table 1.** Primer sequence in this study.

Target gene	Forward (5’−3’)	Reverse (5’−3’)
*GAPDH*	GATGGGCGTGAACCATGAGA	AGCCACCACCTCTCCAGTATTATCC
*ACTB*	TGGACTTCGAGCAAGAGATG	GATCTTGATCTTCATGGTGCT
*STAT1*	GCGGCAGAATTCCGACACCTGCAAC	AGCTGGCTGACGTTGGAGATCACCAC
*Muc2*	GGCTGCTCATTGAGAGGAGT	ACAGCCTCGACATTTCCCTT
*IL6*	TGGCAGAAAA AGACGGATGC	TTGTGCTTCCAGATGCCGTCAG
*IFN-α*	CTGCTGCCTGGAATGAGAGCC	TGACACAGGCTTCCAGGTCCC
*IFN-λ1*	CCACGTCGAACTTCAGGCTT	ATGTGCAAGTCTCCACTGGT
*ISG15*	GGTGAGGAACGACAAGGGTC	GGCTTGAGGTCATACTCCCC
*SVA-N*	TTCGTGCTCCCTTGGAACTA	GTTAGGGCGGGGGATAAAGG

### Statistical analysis

The data from triplicate experiments were analyzed using GraphPad Prism version 8.0 software and are presented as the means ± standard deviations (SDs). One-way analysis of variance (ANOVA) was employed to determine the significance of differences between groups. The variance was considered significant when the *p* value was *p < 0.05, **0.01 < P < 0.05, ***p < 0.01, ****p < 0.001.

## Result

### Establishment of apical-out porcine intestinal organoids culture system

**Figure 1.**
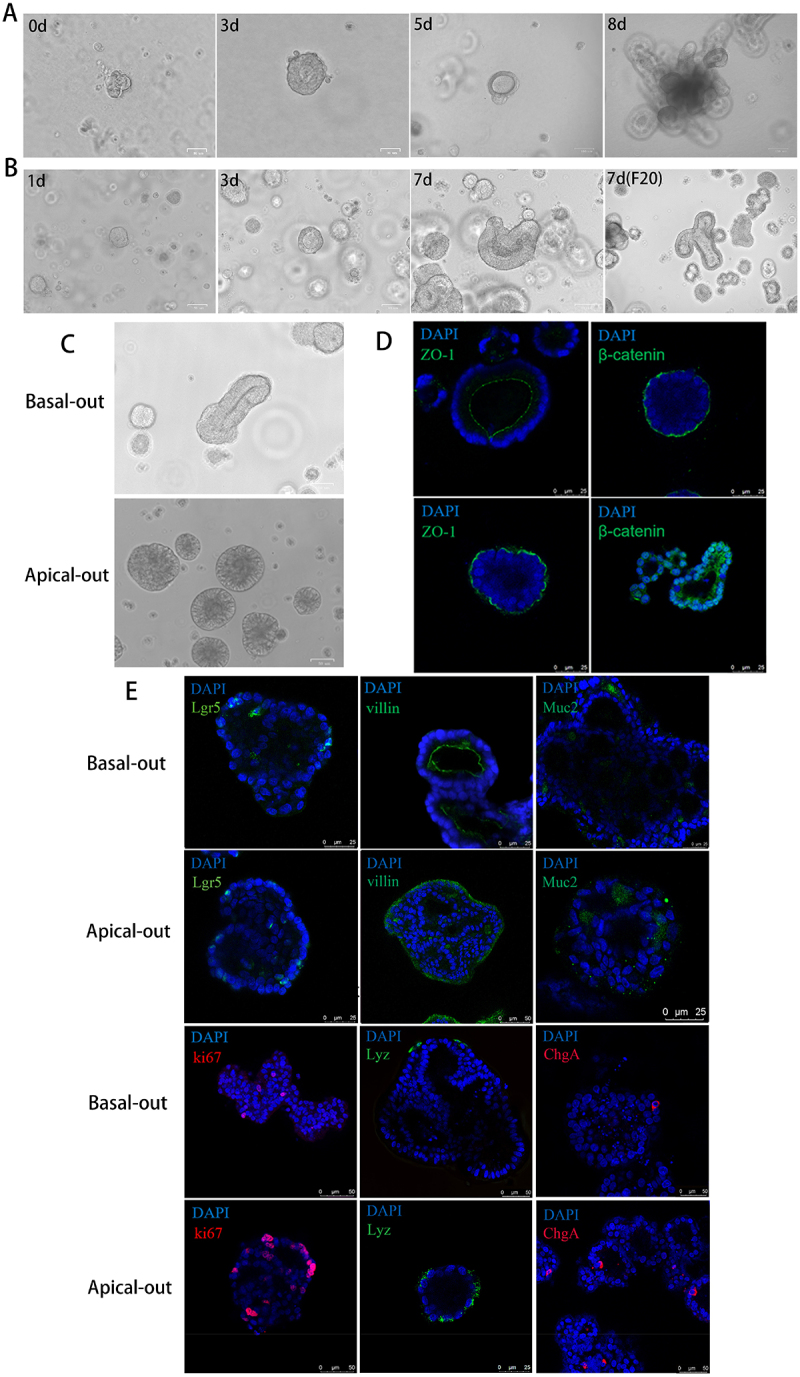
Establishment of apical-out porcine intestinal organoids culture system. (A) The isolation and culture of intestinal crypt stem cell-derived organoids from the jejunum of 7-day-old piglets. The images illustrate the cellular morphology of primary porcine intestinal organoids at days 0, 3, 5 and 8. (B) Porcine intestinal organoids can be successfully resuscitated after cryopreservation and can be continuously passaged and cultured for up to 20 generations. The initial three images depict the cellular morphology of frozen porcine intestinal organoids on day 1, 3, and 7, respectively, following the resuscitation of the cultured organoids. The final image shows the cellular morphology of the 20th generation of porcine intestinal organoids culture over a seven-day period. (C) Removing the Matrigel and separating the organoids was performed on the third day of culture, and Y-27632 was added to the growth medium to induce polarity reversal. The cells of porcine intestinal organoids, cultured for three days and then three days in suspension, were observed under a microscope. (D) Immunofluorescence staining was conducted for the apical protein marker ZO-1 and the basal protein β-catenin. The staining results of the organoids were observed under a confocal microscope to confirm the reversal polarity. (E) To characterize the cell types present in the growing/apical-out porcine intestinal organoids, immunofluorescence was performed on intestinal stem cells (Lgr5), enterocytes (Villin), goblet cells (Muc2), proliferating cells (Ki67), Paneth cells (Lyz), and enteroendocrine cells (ChgA).

To gain insights into the infection pattern of SVA on the intestinal of piglets, we have established apical-out porcine intestinal organoids, which offer a valuable platform for investigating pathogen-host interactions within the intestine. Crypt stem cells were isolated from porcine jejunal segments, and intestinal crypt stem cells exhibited proliferation and differentiation with time in culture. Following an eight-day period, jejunal crypts grew into 3D structures with crypt-like protrusions (Figure 1(A)). Cryopreserved material followed similar growth kinetics even after 20 passages. (Figure 1(B)).

Subsequently, we exposed the apical side of epithelial cells to viral particles and enhanced viral attachment to the organoids by reversing polarity. On the third day of the polarity reversal, apical-out porcine intestinal organoids were obtained, the cavity structure of the apical-out porcine intestinal organoids was not readily discernible upon microscope examination. The cell structure was compact, regular in shape, and smaller in size compared to that of the differentiated non-apical-out porcine intestinal organoids (Figure 1(C)). To further confirm the polarity inversion of the porcine intestinal organoids, immunofluorescence staining was conducted on the apical protein marker ZO-1 and the basal protein β-catenin. The organoid staining results were then observed under a confocal microscope. The results indicated a shift in the expression of ZO-1 shifted from the inner lumen to the outer membrane, accompanied by an increase in β-catenin expression at the tubular lumen of the organoids following polarity inversion, in comparison to the organoids cultured without inversion. These findings confirmed the inversion of the polarity of the organoids, with the apical surface now oriented outward (Figure 1(D)).

To ascertain the consistency of the cell subpopulations present in the porcine intestinal organoids prior to and following the flipping of porcine intestinal epithelial cells, immunofluorescence assays were conducted on intestinal stem cells (Lgr5), enterocytes (Villin), goblet cells (Muc2), proliferating cells (Ki67), Paneth cells (Lyz), and enteroendocrine cells (ChgA). These assays served to confirm the presence of the cell types described above. The results demonstrated the presence of all six cell types of cells in the porcine intestinal organoids, which is consistent with the intestinal epithelial cell lineage of pigs. Additionally, the apical-out porcine intestinal organoids exhibited a similar cellular composition to that observed prior to polarity inversion (Figure 1(E)). These findings indicate that apical-out porcine intestinal organoids comprising intestinal stem cells, enterocytes, goblet cells, proliferating cells, Paneth cells and enteroendocrine cells were cultured and obtained.

### Proliferation of SVA in apical-out porcine intestinal organoids

**Figure 2.**
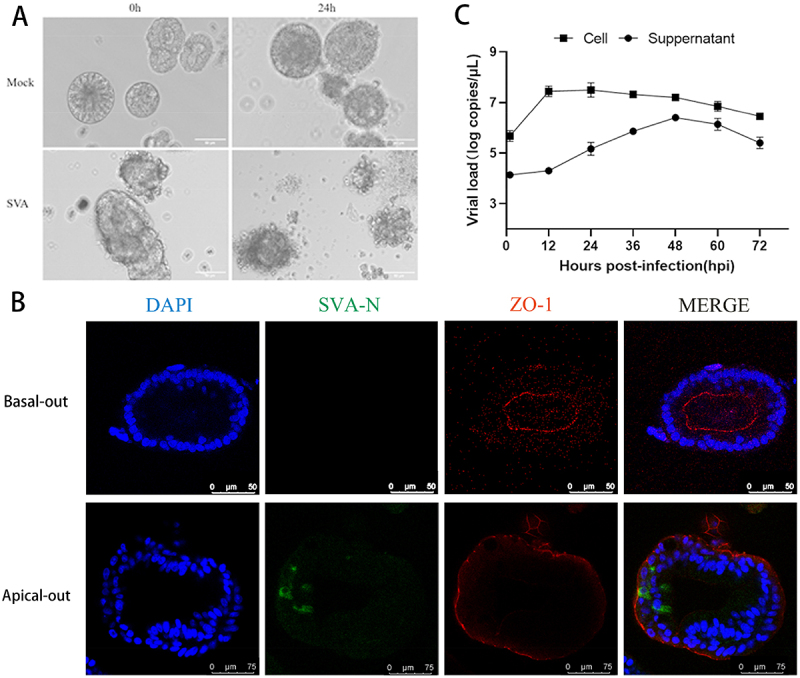
Proliferation of SVA in apical-out porcine intestinal organoids. (A) After the adsorption of the SVA viral solution with MOI of 6 for a period of 2 hours, apical-out porcine intestinal organoids were infected and the cytopathic effects of SVA infected apical-out porcine intestinal organoids were observed over a 24-hour period. (B) Simultaneous immunofluorescence was conducted on apical-out and basal-out porcine intestinal organoids infected for 12 hours using mouse anti-SVA-N protein polyclonal antibodies and the apical protein marker ZO-1 antibody. (C) Precipitates and supernatants from infected cells at various time points were collected and the viral copy number was calculated using RT-qPCR. The error bars represent the standard deviations of the technical triplicates, with *n* = 3 per group.

To investigate whether SVA can infect the apical-out porcine intestinal organoids and determine if these organoids exhibit damage similar to that observed in the small intestine epithelium in vivo, we infected the apical-out porcine intestinal organoids with an SVA viral solution at MOI of 6. After 24 h of SVA infection, apical-out porcine intestinal organoids lose their intact, tightly-organized capsule structure and disintegrate (Figure 2(A)). To further confirm the SVA infection in the apical-out porcine intestinal organoids, we performed immunofluorescence on apical-out and basal-out porcine intestinal organoids infected for 12 hours. The results showed that the N protein of SVA localized in the apical-out porcine intestinal organoids, and expressing ZO-1 in the outer membrane (Figure 2(B)).

We collected cells and culture supernatants from SVA-infected organoids at different time points and used RT-qPCR to quantify viral copy numbers. The results demonstrated that SVA exhibited the highest viral copy number at 12 hours post-infection (hpi), with an approximately 60-fold increase from 4.9 × 10^5^ copies/μL to 2.9 × 10^7^ copies/μL at 12 hpi compared with 1 hpi. In addition, the viral load in the culture supernatant kept increasing with incubation time, peaking at 48 hpi (Figure 2(C)). These results indicate that SVA successfully infected apical-out porcine intestinal organoids and established effective viral infection.

### Identification of cellular subpopulations in SVA-infected organoids at different time points

**Figure 3.**
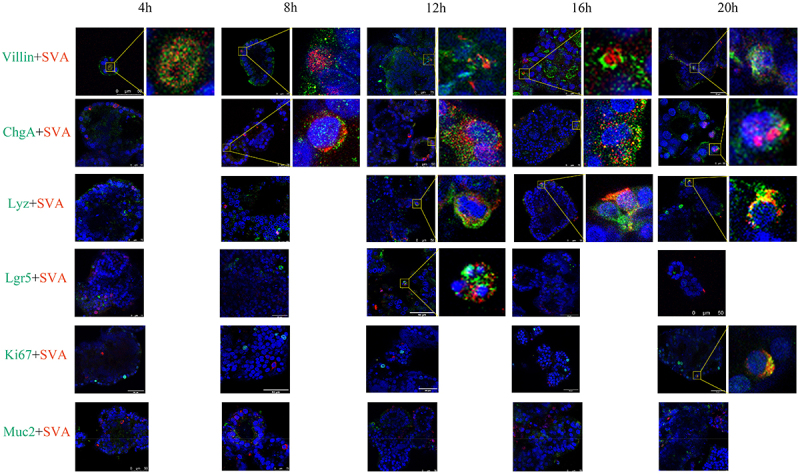
Identification of cellular subpopulations in organoids infected with SVA at various time points. The co-localization of the SVA-N protein with enterocytes, enteroendocrine cells, Paneth cells, intestinal stem cells, proliferating cells, and goblet cells were evaluated through double immunofluorescence labeling at 4 hpi, 8 hpi, 12 hpi, 16 hpi, and 20 hpi, respectively.

The objective of this study is to gain further insight into the infectious properties of SVA in apical-out porcine intestinal organoids. The types of SVA-infected cells were examined at various time points. Double immunofluorescence labeling was employed to assess the co-localization of intestinal stem cells (Lgr5), enterocytes (Villin), goblet cells (Muc2), proliferating cells (Ki67), enteroendocrine cells (ChgA) and Paneth cells (Lyz) as well as SVA-infected cells, at 4 hpi, 8 hpi, 12 hpi, 16 hpi, and 20 hpi. The co-localization of enterocytes with the SVA-N protein was observed as early as 4 hpi, with consistent co-localized fluorescence detected at 8 hpi, 12 hpi and 16 hpi, and 20 hpi. These results indicate that SVA has a preferential infection of enterocytes, which appear to be the first cell type affected. We observed significant co-localization of SVA-N protein with ChgA of enteroendocrine cells at 8 hpi, 12 hpi, 16 hpi and 20 hpi. This finding suggests that SVA is able to infect enteroendocrine cells, albeit later than enterocytes. Co-localization of SVA-N protein with Lyz of Paneth cells and Lgr5 of intestinal stem cells was observed at 12 hpi, as well as co-localization of SVA-N protein with Ki67 of proliferating cells at 20 hpi, suggesting that these Paneth and intestinal stem cells, as well as proliferating cells, may also be susceptible to infection by SVA after infection of enterocytes and enteroendocrine cells. Notably, no co-localization was observed between Muc2, a marker protein of goblet cells, and the SVA-N protein at any of the five time points assessed in the co-localization assay (Figure 3). The results indicate that SVA is capable to infecting enterocytes, enteroendocrine cells, Paneth cells, intestinal stem cells, and proliferating cells. However, it was not detected in goblet cells. In conclusion, our findings indicate that SVA can infect various subpopulations of cells within intestinal organoids. The order of cells infected by SVA in intestinal organoids is as follows: enterocytes, enteroendocrine cells, Paneth cells, intestinal stem cells, and proliferating cells. Of these, enterocytes appear to be the primary target cells for SVA infection.

### Alteration of SGs pattern in SVA-infected apical-out porcine intestinal organoids

**Figure 4.**
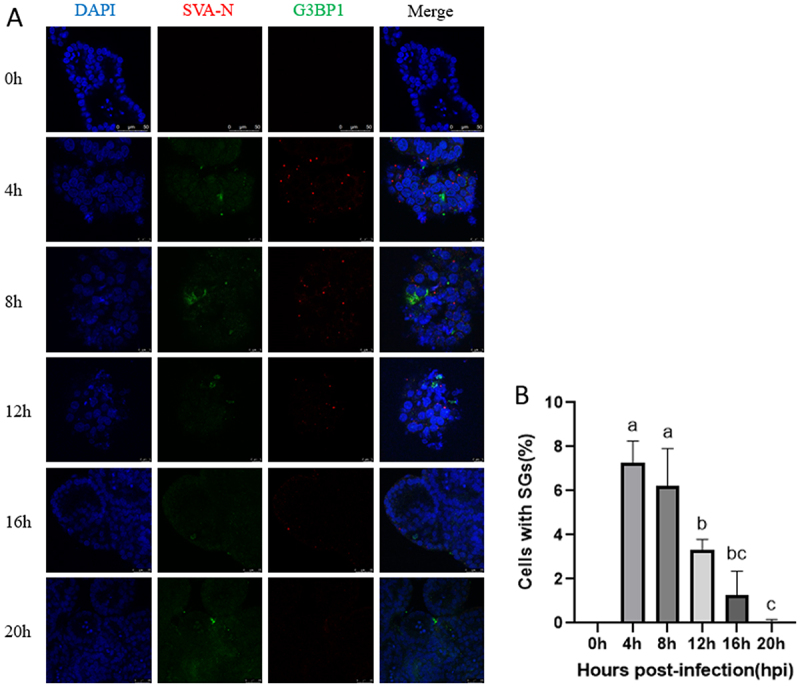
Detection of SGs in apical-out porcine intestinal organoids as a model of SVA infection. (A) The formation of SGs induced by SVA infection in organoids at time points of 0hpi, 4hpi, 8hpi, 12hpi, 16hpi, and 20hpi was assessed through indirect immunofluorescence detection of G3BP1 and SVA-N proteins. (B) The percentage of cells containing SGs was calculated in three independent experiments, with *n* = 3 per group. Different letters (a vs. b, a vs. c) indicate significant differences (*p* < 0.05); the same letter indicates non-significant differences (*p* > 0.05): one-way ANOVA was employed to determine the significance of differences between groups.

SGs are defensive structures produced by cells in response to external stimuli and viral infections. To ascertain whether SVA infection of porcine intestinal organoids results in the formation of SGs, apical-out porcine intestinal organoids were infected with SVA at various time points (0 hpi, 4 hpi, 8 hpi, 12 hpi, 16 hpi, and 20 hpi). An indirect immunofluorescence assay was employed to ascertain the localization of G3BP1, a core component of SGs, to the SVA-N protein, with a view to determine whether SVA infection is responsible for SGs formation. The results indicated that SGs began to appear in large numbers 4 hours after infection and exhibited punctate aggregation in cells. These punctate aggregates gradually diminished with the duration of infection. No punctate aggregates were observed 20 hours of viral infection (Figure 4(A)). We performed three replicated experiments, each with five fields of view selected at the top, bottom, left, right, and center. Then, we performed quantitative analysis of fluorescence images based on gray values using Image J software, showing the percentage of SG observed in apical-out porcine intestinal organoids (Figure 4(B)). These findings suggest that SVA induces the formation of transient SGs.

### Transcriptional changes in antiviral-related genes in apical-out porcine intestinal organoids as a model of SVA infection

**Figure 5.**
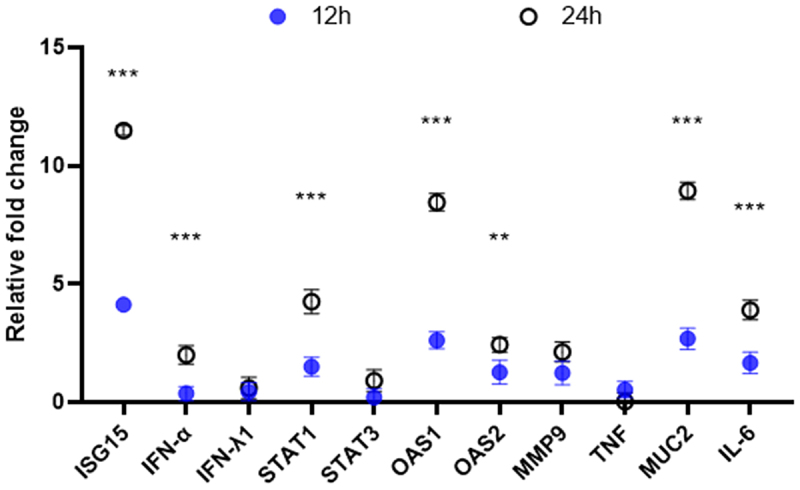
The relative transcript levels of immune cytokines were quantified following the SVA infection of organoids. Relative gene expression levels of ISG15, IFN-α, INF-λ1, STAT1, STAT3, OAS1, OAS2, MMP9, TNF, MUC2, and IL-6 at 12 hpi and 24 hpi are detected by RT-qPCR and shown (mean±sd). Significant differences between the 12hpi and 24hpi groups are indicated by asterisks. *p < .05, **0.01 < P < 0.05, ***p < .01, ****p < .001: one-way ANOVA was employed to determine the significance of differences between groups.

Enterovirus infection of intestinal tissues elicits an immune response and induces changes in various antiviral-related cytokines. To investigate the corresponding changes in cytokines by SVA infection of apical-out porcine intestinal organoids, a series of cytokines were measured by RT-qPCR in this study. The relative changes in mRNA levels of these cytokines were calculated after 12 and 24 hours of SVA infection. The results demonstrated that the transcriptional alternations in antiviral-related genes were more pronounced after 24 hours of infection compared to 12 hours. Additionally, IFN-α, a pivotal element in the innate immune response, exhibited a notable up- regulation, reaching a 9-fold increase. Furthermore, the interferon-stimulated genes ISG-15, OAS1, and OAS2 exhibited a notable elevation, with an 11-fold increase in ISG-15 and an 8.4-fold increase in OAS1. Additionally, the signal transducer and transcriptional activator STAT1, the cytokine IL-6, and the mucosal immune-related gene Muc2 were also significantly upregulated (Figure 5). These findings indicate that multiple antiviral-associated immune response factors can be induced during SVA infection of apical-out porcine intestinal organoids, which may have an impact on the infection process of SVA.

## Discussion

Senecavirus A (SVA) is a pathogenic picornavirus that can cause blistering and ulcerative disease in pigs. It was discovered in recent years, and its clinical presentation is strikingly similar to the symptoms of foot-and-mouth disease and porcine blistering disease. The majority of SVA-infected piglets have a documented history of diarrhea. A considerable proportion of these piglets present with subcutaneous or mesenteric edema [26]. Histological analysis of the affected intestines reveals atrophy and fusion of intestinal villi, in addition to vacuolization of superficial epithelial cells. Furthermore, the presence of SVA can be confirmed in these lesions. This indicates that SVA has the potential to induce gastrointestinal disease in neonatal piglets by damaging intestinal cells [1]. The current lack of in vitro SVA infection of intestinal cell models restricts the investigation of the mechanisms associated with SVA infection of the intestine.

Therefore, we initially established a porcine intestinal organoid model that more closely resembles the in vivo intestinal cell composition, structure, and physiological state. Subsequently, we employed apical-out porcine intestinal organoids, thereby achieving translocation of the functional region and enabling direct interaction with SVA. Following the inoculation of SVA into the apical-out porcine intestinal organoids, the formation of cellular lesions and the proliferation of the virus within the cells were observed. In the apical-out porcine intestinal organoids, the peak of viral copy number was rapidly reached 12 hours after SVA infection, with the highest viral titer of approximately 2.9 × 10^7^ copies/μL. The growth curves of apical-out porcine intestinal organoids exhibited comparable replication kinetics following infection with SVA, as observed in PK-15 cells [27]. In contrast, IBRS-2 cells in vitro may require a longer period of time to reach the same viral titer after infection with SVA. This may be attributed to the biological properties of the cells and their susceptibility to the virus [28,29].

Following the infection of apical-out porcine intestinal organoids with SVA, the virus infected enterocytes after 4 hpi, enteroendocrine cells after 8 hpi, Paneth cells and intestinal stem cells after 12 hpi and ultimately proliferating cells after 20 hpi. It was therefore postulated that the virus might further trigger infection of other cell types by invading enterocytes and thus destroying the structure. This hypothesis is also in line with the viewpoint of Burrows R et al. that SVA triggers infection in vivo through direct contact with the skin or intestinal epithelium after entering the body [30], resulting in a loss of barrier function and allowing the virus to reach deeper enteroendocrine cells and Paneth cells. The potential for infection of intestinal stem cells and proliferating cells may facilitate synchronous transfer of SVA to daughter cells concomitant with cell division, creating a persistent infection. Such a process could result in the rapid propagation of the virus in the cell populations of porcine intestinal organoids. This mechanism may underpin persistent infection in piglets, as evidenced in apical-out porcine intestinal organoids [31].

In response to external stimuli and viral infection, cells produce SGs, a defensive measure that appears early in the course of viral infection. SGs are mRNA storage sites that regulate mRNA translation. The G3BP1-induced SGs increased rapidly and reached a peak 4 hours after SVA virus infection of apical-out porcine intestinal organoids. Thereafter, they began to decline gradually and almost disappeared at 20 hpi. This finding is consistent with previous reports that SVA induces transient SGs formation in a PKR- and eIF2α-phosphorylation-dependent manner during the early stage of infection of HEK 293T cells. Additionally, SVA inhibits SGs formation via 3C proteins in the mid- to late stage of infection and disrupts the eIF4GI-G3BP1 interactions, thereby inhibiting the generation of SGs [24]. The proliferation cycle of the SVA virus is consistent with the observed peak of viral proliferation occurring 12 hpi, which is significantly later than the time of mass production of stress particles. The virus exhibited rapid proliferation over a period of 12 hpi to 24 hpi. This phenomenon may be attributed to the fact that the retention of translation initiation factors in SGs inhibits the expression of antiviral factors (e.g. interferon) without affecting the protein expression of the virus [32]. Furthermore, the function of pivotal proteins such as G3BP1 in SGs commenced its inhibition, and SVA interfered with the defense mechanism of SGs through its specific proteins, thus promoting its own replication and transmission [24,33,34]. Viral copy numbers gradually declined from 24 hpi, likely due to the dissipation of antiviral factor-mediated inhibition in SGs and the concurrent activation of innate immunity [32]. The results demonstrated that numerous antiviral-related factors of the immune response underwent significant alterations at 24 hpi. In particular, Viral copy number showed a gradual decline from 24 hpi, which may be related to the interferon-stimulating factors ISG-15 and OAS1, the interferon IFN-α, the signal transducer and activator of transcription STAT1, the cytokine IL-6, and the mucosal barrier-related gene Muc2 were significantly upregulated. Although it has been demonstrated that SVA can evade the immune system by inhibiting type I interferon (IFN-α/β) [35], this result was presented based on HEK293T and PK-15 cells, whereas the cells involved in the present study were derived from intestinal tissues.

In conclusion, the present study employed apical-out porcine intestinal organoids culture system to investigate the susceptibility and proliferative properties of SVA to intestinal cells in vitro. This investigation initially elucidates the infection pattern of SVA in intestinal epithelial cells and provides mechanistic insight into SVA-host interactions in cells with low permissiveness. It provides mechanistic insight into the disruption of intestinal homeostasis by SVA infection and establishes a foundation for targeted strategies to suppress viral replication and prevent secondary infections by preserving epithelial integrity and regulating immune responses.

## Data Availability

The data that support the findings of this study are openly available in Mendeley at https://doi.org/10.17632/mrjsszgwjv.1 (DOI: 10.17632/mrjsszgwjv.1). ARRIVE guidelines: The study adhered to the ARRIVE guidelines and uploaded a complete checklist in supplemental material.

## References

[1] Leme RA, Oliveira TES, Alfieri AF, et al. Pathological, immunohistochemical and molecular findings associated with Senecavirus A-induced lesions in neonatal piglets. J Comp Pathol. 2016;155(2–3):145–13. doi: 10.1016/j.jcpa.2016.06.011 27473601

[2] Reddy PS, Burroughs KD, Hales LM, et al. Seneca Valley virus, a systemically deliverable oncolytic picornavirus, and the treatment of neuroendocrine cancers. J Natl Cancer Inst. 2007;99(21):1623–1633. doi: 10.1093/jnci/djm198 17971529 PMC5261858

[3] Munday BL, Ryan FB. Vesicular lesions in swine–possible association with the feeding of marine products. Aust Vet J. 1982;59(6):193. doi: 10.1111/j.1751-0813.1982.tb16008.x 6301415

[4] Vannucci FA, Linhares DCL, Barcellos D, et al. Identification and complete genome of Seneca Valley virus in vesicular fluid and sera of pigs affected with idiopathic vesicular disease, Brazil. Transbound Emerg Dis. 2015;62(6):589–593. doi: 10.1111/tbed.12410 26347296

[5] Zhao XY, Wu QW, Wu ZX, et al. Isolation and characterization of the first porcine Seneca Valley virus in China. Chin J Preventative Vet Med. 2016;38(11):839–843.

[6] Hales LM, Knowles NJ, Reddy PS, et al. Complete genome sequence analysis of Seneca Valley virus-001, a novel oncolytic picornavirus. J Gener Virol. 2008;89(5):1265–1275. doi: 10.1099/vir.0.83570-0 18420805

[7] Luo TX. Isolation and characterization of porcine Senecavirus a (SVA) HN16 and its biological properties [master thesis]. South China Agricultural University; 2018.

[8] Oliveira TES, Michelazzo MMZ, Fernandes T, et al. Histopathological, immunohistochemical, and ultrastructural evidence of spontaneous Senecavirus A-induced lesions at the choroid plexus of newborn piglets. Sci Rep. 2017;7(1):16555. doi: 10.1038/s41598-017-16407-0 29185462 PMC5707367

[9] Guo B, Piñeyro PE, Rademacher CJ, et al. Novel senecavirus a in swine with vesicular disease, United States, July 2015. Emerg Infect Dis. 2016;22(7):1325. doi: 10.3201/eid2207.151758 27314645 PMC4918180

[10] Bracht AJ, O’Hearn ES, Fabian AW, et al. Real-time reverse transcription PCR assay for detection of Senecavirus a in swine vesicular diagnostic specimens. PLOS ONE. 2016;11(1):e0146211. doi: 10.1371/journal.pone.0146211 26757142 PMC4710529

[11] Chen M, Zhang X, Kong F, et al. Senecavirus a induces mitophagy to promote self-replication through direct interaction of 2C protein with K27-linked ubiquitinated TUFM catalyzed by RNF185. Autophagy. 2024;20(6):1286–1313. doi: 10.1080/15548627.2023.2293442 38084826 PMC11210902

[12] Fujii M, Sato T. Somatic cell-derived organoids as prototypes of human epithelial tissues and diseases. Nat Mater. 2021;20(2):156–169. doi: 10.1038/s41563-020-0754-0 32807924

[13] Saxena K, Blutt SE, Ettayebi K, et al. Human intestinal enteroids: a new model to study human rotavirus infection, host restriction, and pathophysiology. J Virol. 2016;90(1):43–56. doi: 10.1128/jvi.01930-15 26446608 PMC4702582

[14] Yin Y, Bijvelds M, Dang W, et al. Modeling rotavirus infection and antiviral therapy using primary intestinal organoids. Antiviral Res. 2015;123:120–131. doi: 10.1016/j.antiviral.2015.09.010 26408355

[15] Williamson IA, Arnold JW, Samsa LA, et al. A high-throughput organoid microinjection platform to study gastrointestinal microbiota and luminal physiology. Cell Mol Gastroenterol Hepatol. 2018;6(3):301–319. doi: 10.1016/j.jcmgh.2018.05.004 30123820 PMC6092482

[16] Afting C, Walther T, Drozdowski OM, et al. DNA microbeads for spatio-temporally controlled morphogen release within organoids. Nat Nanotechnol. 2024;19(12):1849–1857. doi: 10.1038/s41565-024-01779-y 39251862 PMC11638066

[17] Li L, Fu F, Guo S, et al. Porcine intestinal enteroids: a new model for studying enteric coronavirus porcine epidemic diarrhea virus infection and the host innate response. J Virol. 2019;93(5):e01682–18. doi: 10.1128/jvi.01682-18 30541861 PMC6384061

[18] Usui T, Sakurai M, Umata K, et al. Preparation of human primary colon tissue‐derived organoid using air liquid interface culture. Curr Protoc Toxicol. 2018;75(1):.22.6.1–.22.6.7. doi: 10.1002/cptx.40 29512123

[19] Sabapaty A, Lin PY, Dunn JCY. Effect of air–liquid interface on cultured human intestinal epithelial cells. FASEB Bioadv. 2024;6(2):41. doi: 10.1096/fba.2023-00132 38344411 PMC10853644

[20] Co JY, Margalef-Catala M, Li X, et al. Controlling epithelial polarity: a human enteroid model for host-pathogen interactions. Cell Rep. 2019;26(9):2509–2520.e4. doi: 10.1016/j.celrep.2019.01.108 30811997 PMC6391775

[21] Li Y, Yang N, Chen J, et al. Next-generation porcine intestinal organoids: an apical-out organoid model for swine enteric virus infection and immune response investigations. J Virol. 2020;94(21):e01006–20. doi: 10.1128/jvi.01006-20 32796075 PMC7565635

[22] Zhao K, Zhang S, Liu X, et al. The game between host antiviral innate immunity and immune evasion strategies of senecavirus A - A cell biological perspective. Front Immunol. 2022;13:1107173. doi: 10.3389/fimmu.2022.1107173 36618383 PMC9813683

[23] Alam U, Kennedy D. Rasputin a decade on and more promiscuous than ever? A review of G3BPs. Biochim Biophys Acta Mol Cell Res. 2019;1866(3):360–370. doi: 10.1016/j.bbamcr.2018.09.001 30595162 PMC7114234

[24] Wen W, Zhao Q, Yin M, et al. Seneca Valley virus 3C protease inhibits stress granule formation by disrupting eIF4GI-G3BP1 interaction. Front Immunol. 2020;11:577838. doi: 10.3389/fimmu.2020.577838 33133097 PMC7550656

[25] Gao YY, Guo CY, Jiang XL, et al. Construction of G3BP1-/-293T cell line and its effect on Senecavirus a replication. Chin Vet Sci. 2023;53(8):977–982.

[26] Niedbalski W, Fitzner A. Senecavirus a: an emerging pathogen causing vesicular disease in pigs. 2019. doi: 10.1111/tbed.12430

[27] Gao H, Xiang Z, Ge X, et al. Comparative proteomic analysis reveals Mx1 inhibits Senecavirus a replication in PK-15 cells by interacting with the capsid proteins VP1, VP2 and VP3. Viruses. 2022;14(5):863. doi: 10.3390/v14050863 35632606 PMC9147370

[28] Tao Q, Zhu L, Xu L, et al. The construction and immunogenicity analyses of a recombinant pseudorabies virus with senecavirus a VP2 protein coexpression. Microbiol Spectr. 2023;11(2):e0522922. doi: 10.1128/spectrum.05229-22 36976021 PMC10100989

[29] Jia M, Sun M, Tang YD, et al. Senecavirus a entry into host cells is dependent on the cholesterol-mediated endocytic pathway. Front Vet Sci. 2022;9:840655. doi: 10.3389/fvets.2022.840655 35498725 PMC9040607

[30] Burrows R, Mann JA, Goodridge D. Swine vesicular disease: virological studies of experimental infections produced by the England/72 virus. Epidemiol Infect. 1974;72(1):135–143. doi: 10.1017/s0022172400023305 PMC2130259 4522243

[31] Rambani K, Vukasinovic J, Glezer A, et al. Culturing thick brain slices: an interstitial 3D microperfusion system for enhanced viability. J Neurosci Methods. 2009;180(2):243–254. doi: 10.1016/j.jneumeth.2009.03.016 19443039 PMC2742628

[32] Song Y, Mugavero J, Stauft CB, et al. Dengue and Zika virus 5’ untranslated regions harbor internal ribosomal entry site functions. MBio. 2019;10(2):e00459–19. doi: 10.1128/mbio.00459-19 30967466 PMC6456755

[33] Sun D, Kong N, Dong S, et al. 2AB protein of Senecavirus a antagonizes selective autophagy and type I interferon production by degrading LC3 and MARCHF8. Autophagy. 2022;18(8):1969–1981. doi: 10.1080/15548627.2021.2015740 34964697 PMC9450971

[34] Yang X, Liu R, Du Y, et al. CircRNA_8521 promotes senecavirus a infection by sponging miRNA-324 to regulate LC3A. Vet Res. 2024;55(1):43. doi: 10.1186/s13567-024-01291-0 38581048 PMC10996121

[35] Xue Q, Liu H, Zhu Z, et al. Seneca Valley virus 3Cpro abrogates the IRF3- and IRF7-mediated innate immune response by degrading IRF3 and IRF7. Virology. 2018;518:1–7. doi: 10.1016/j.virol.2018.01.028 29427864

